# Sex-biased phenotypic plasticity affects sexual dimorphism patterns under changing environmental conditions

**DOI:** 10.1038/s41598-024-51204-6

**Published:** 2024-01-09

**Authors:** Giulia Cordeschi, Daniele Canestrelli, Daniele Porretta

**Affiliations:** 1 Department of Environmental Biology, Sapienza University of Rome, Via Dei Sardi 70, Rome, Italy; 2 Department of Biology and Ecology, Tuscia University, Largo Dell’Università S.N.C., Viterbo, Italy

**Keywords:** Evolutionary ecology, Entomology

## Abstract

Sexual dimorphism is almost ubiquitous in animals. A common pattern observed across multiple taxa involves differences in development time (sexual bimaturism) and body size (sexual size dimorphism) between conspecific males and females. Furthermore, a strict association of dimorphism at these traits has been documented in several taxa, where the sex showing shorter development time also has a smaller body size than the other sex. Growth and development are strongly dependent on environmental conditions during individual life-cycle in ectotherms, inducing considerable phenotypic plasticity. However, how phenotypic plasticity affects the association between sexual dimorphism in development time and body size remains unclear. Here, we tracked development time, body size, and body mass throughout the ontogeny of the mosquito *Aedes mariae*. The larval development of this species is strictly linked to Mediterranean Sea rock-pools, whose highly variable environmental conditions over minimal time frames make this organism-environment system ideal for exploring plasticity-led eco-evolutionary processes. We found differential plasticity between males and females, dissolving the link between dimorphism in development time and body size under increasing temperature and decreasing salinity conditions. These findings contrast with the current hypotheses proposed to explain the origin of the association between sexual bimaturism and sexual size dimorphism, highlighting the condition dependence of sexual dimorphism patterns and the need to consider phenotypic plasticity in future studies on their evolution.

## Introduction

Phenotypic differences between males and females of the same species are widespread across the animal kingdom, and they are exhibited in a myriad of traits, including morphological, behavioural and life-history traits^1^. Well-known and iconic examples like those found among birds of paradise^2^ or stag beetles^3^, as well as some less conspicuous but intriguing cases, like sexually dimorphic traits in our own species, have fuelled intense scientific curiosity, research, and discussions around the origin, evolutionary underpinnings and ecological implications of sexual dimorphisms^4,5^. Sexual bimaturism is a form of sexual dimorphism consisting of different development times between the two sexes^6,7^. In insects, sexual bimaturism is widespread and takes the form of a sex difference in the timing of adult emergence: protandry, if adult males appear before adult females, and protogyny, if the opposite is the case^7–9^. Interestingly, protandry has been frequently associated with female-biased sexual size dimorphism^9–13^, but what drives this association in nature is still an entirely open question surrounding the evolution of sexual dimorphism.

At least two hypotheses have been proposed to explain cases of association between protandry and female-biased sexual size dimorphism^9,14^. First, protandry evolved under selection for early male maturation (called the mating opportunity hypothesis). Consequently, shortening male development time would lead to a smaller male size and a female-biased sexual size dimorphism^15^. This hypothesis was invoked to explain sexual bimaturism in organisms with short life span and discrete generations, where males maturing before females may gain a fitness advantage by increasing their mating opportunities^10^. Second, protandry would be a by-product of selection for a large female size (the constraint hypothesis). In insects, female body size has been linked to fecundity and egg production, so the fitness advantage of larger size might be greater for females than males^16^. If females and males grow at similar rates, selection for large female body size would, thus, require longer female growth, with protandry evolving as an indirect consequence^17^.

Development time and body size are two life-history traits genetically determined but also widely plastic, and sexes might differ in their sensitivity to environmental conditions during development^18^. In ectotherms, growth and development depend strongly on environmental variables such as diet quantity and quality or temperature, inducing considerable phenotypic plasticity ^9,19,20^. For example, in several insect species, females extend their development time more than males under unfavourable conditions and show greater plasticity than males in body size^19^. Several studies showed a positive correlation between sex-specific plasticity of a trait and sexual dimorphism in the same trait^19,21–23^. However, how sex-specific plasticity affects association and multiple trait covariation remains unclear.

Interestingly, differences in phenotypic plasticity between sexes could weaken the two hypotheses described above and the predicted relationship between protandry and female-biased sexual size dimorphism^24^. According to the mating opportunity hypothesis, the difference in development time between males and females should be largely insensitive to environmental conditions experienced during juvenile growth^8,9,25,26^. Consequently, differences between sexes in the plasticity of development time would be at odds with this hypothesis. At the same time, according to the constraint-hypothesis, males and females should grow at similar rates, with no plasticity in growth trajectories. Thus, differences in plasticity in development time and body size between sexes could dissolve the association between protandry and female-biased size dimorphism^19^.

**Figure 1:**
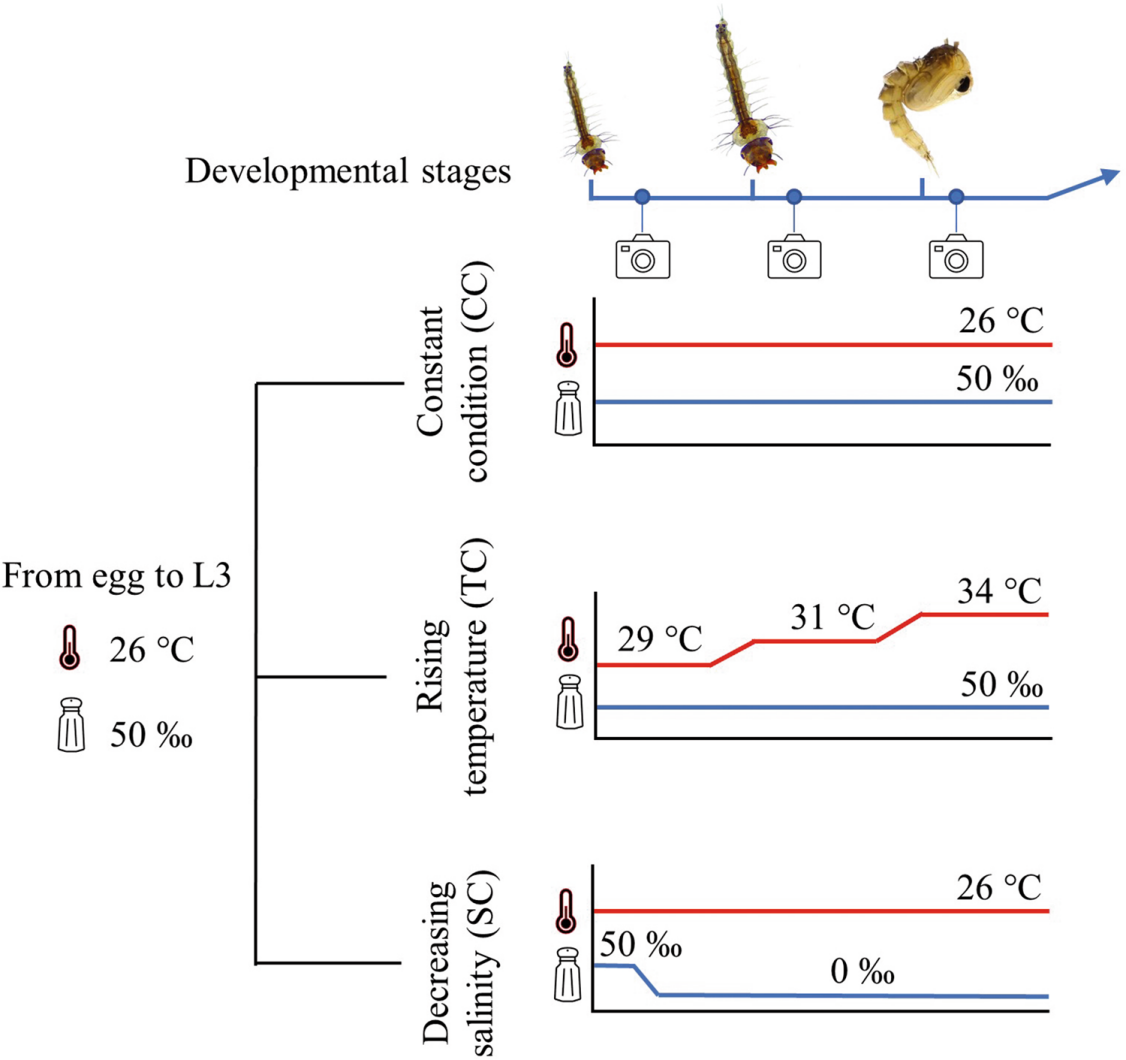
Experimental design. All individuals from the egg to third instar larvae were kept at 26 °C and 50 ‰ salinity. Third instar larvae were randomly placed into developmental treatments, and trait measures started. In the first treatment, individuals were maintained at constant conditions of temperature (26 ±  °C) and salinity (50‰, 50 g/l) throughout the experiment (CC). In the second treatment, individuals were exposed to rising temperature (TC) as follows: third instar larvae were maintained at 29 °C; fourth instar larvae at 31 °C, and pupae at 34 °C. In the third treatment, individuals were exposed to decreasing salinity (SC) from 50‰ to 0‰. Weight and digital pictures were obtained within two hours from the ecdysis.

In this paper, we investigated in the sea rock-pool mosquito *Aedes mariae* (Sergent and Sergent, 1903) the role of sexual differences in phenotypic plasticity in determining sexual dimorphism. This species is distributed along the western Mediterranean coast, developing in rock pools in the supralittoral zone of coastal habitats^27^. Rock pools along the marine littoral are extremely variable environments, experiencing dramatic fluctuations in temperature and salinity over short timescales^28^ due to rainfall, storms, tides, and evaporation^29^. Environmental temperature is one of the most important abiotic factors influencing insects' physiology, behaviour, ecology and survival^30^ whereas salinity was observed to influence development of individuals of *Ae. mariae* complex species ^31,32^.Therefore, because of the extreme environmental variations, this organism-environment system is ideal for exploring phenotypic plasticity. Moreover, female-biased sexual size dimorphism and protandrous sexual bimaturism have been described in mosquito species^33,34^. Here, we performed microcosm experiments manipulating temperature and salinity and measured the individual plastic response in development time and body size aiming to: (i) determine if sexual bimaturism and/or female-biased sexual size dimorphism occur and when they arise during development; (ii) investigate if sex-based differential plasticity in development time and body size occurs; (iii) examine the effect of phenotypic plasticity on the association between sexual bimaturism and female-biased sexual size dimorphism (Fig. 1).

## Results

### Development time

The development time from L3 to L4 larval instars, from L4 larval instar to pupal stage, from pupal to adult stages and the total development time from L3 larval instar to adult stage were compared between sexes and treatments (i.e. constant conditions, rising temperature, decreasing salinity during individual development) (Table 1). The results of the 3-way ANOVA analysis showed that the model explains a high percentage of the variance (Adjusted R-squared 0.92). Significant effects of treatment (*F*_*2,599*_ = 64.24, *P* < 0.001), sex (*F*_*1,599*_ = 19.24, *P* < 0.001), and stage (*F*_*3,599*_ = 2487.22, *P* < 0.001), as well as treatment * stage (*F*_*6,599*_ = 13.86, *P* < 0.001) and sex * stage (*F*_*3,599*_ = 3.20, *P* = 0.02) interactions were observed (Supplementary Table S1).

**Table 1:** Summary statistics of *Aedes mariae* phenotypic traits. Mean, standard error and sample size (n). L3-L4 is the development time computed from L3 to L4 larval instars; L4-P is the development time from L4 larval instar to pupal stage; P-Ad is the development time from pupal to adult stages; Total means the development time from L3 larval instar to adult stage.

		Constant condition				Rising temperature				Decreasing salinity			
		Female		Male		Female		Male		Female		Male	
		n	Mean ± se	n	Mean ± se	n	Mean ± se	n	Mean ± se	n	Mean ± se	n	Mean ± se
Development time (h)	L3-L4	31	69.74 ± 1.99	39	68.23 ± 2.39	30	53.73 ± 0.67	28	50.43 ± 2.31	37	69.54 ± 2.26	25	65.72 ± 1.93
	L4-P	31	122.45 ± 2.12	38	110.82 ± 1.79	30	92.43 ± 4.94	28	87.14 ± 2.55	37	100.86 ± 1.04	25	95.20 ± 0.96
	P-Ad	15	60.40 ± 2.67	22	61.14 ± 2.08	28	55.89 ± 2.24	14	49.35 ± 1.17	19	62.79 ± 1.95	25	62.92 ± 2.17
	Total	15	216.27 ± 7.19	22	197.55 ± 4.45	28	191.54 ± 6.85	14	186.7 ± 4.09	19	229.95 ± 3.31	22	217.16 ± 4.55
Weight (mg)	L4	15	1.63 ± 0.07	18	1.35 ± 0.06	14	1.46 ± 0.98	13	1.29 ± 0.09	19	1.74 ± 0.10	11	1.45 ± 0.10
	P	16	5.41 ± 0.24	19	3.89 ± 0.12	14	4.29 ± 0.21	13	3.27 ± 0.15	19	6.49 ± 0.13	12	3.85 ± 0.12
Morphometric measures 4th instar (mm)	Head width	15	1.72 ± 0.03	19	1.71 ± 0.02	14	1.61 ± 02	13	1.60 ± 0.02	18	1.75 ± 0.12	11	1.611 ± 0.04
	Thorax width	15	1.98 ± 0.04	19	1.93 ± 0.03	14	1.88 ± 04	13	1.77 ± 0.02	18	2.07 ± 0.03	11	1.86 ± 0.05
	Abdomen width	15	1.04 ± 0.01	19	0.10 ± 0.01	14	0.99 ± 0.01	13	0.94 ± 0.01	18	1.08 ± 0.01	11	0.94 ± 0.02
	Thorax length	15	1.19 ± 0.03	19	1.12 ± 0.03	14	1.17 ± 0.03	13	1.09 ± 0.03	18	1.25 ± 0.03	11	1.14 ± 0.05
	Abdomen length	15	4.23 ± 0.07	19	4.06 ± 0.10	14	4.04 ± 0.08	13	3.82 ± 0.07	18	4.43 ± 0.09	11	4.05 ± 0.09
	Total length	15	6.42 ± 0.14	19	5.10 ± 0.14	14	6.18 ± 0.12	13	5.87 ± 0.12	18	6.54 ± 0.15	11	6.08 ± 0.21
Morphometric measures pupae (mm)	Cephalo-thorax width	16	3.48 ± 0.05	19	3.14 ± 0.03	13	3.24 ± 0.06	12	2.95 ± 0.03	19	3.63 ± 0. 03	12	3.14 ± 0.03
	Cephalo-thorax length	16	4.21 ± 0.070	19	3.75 ± 0.03	13	3.79 ± 0.07	12	3.47 ± 0.03	19	4.39 ± 0.04	12	3.85 ± 0.04

**Figure 2:**
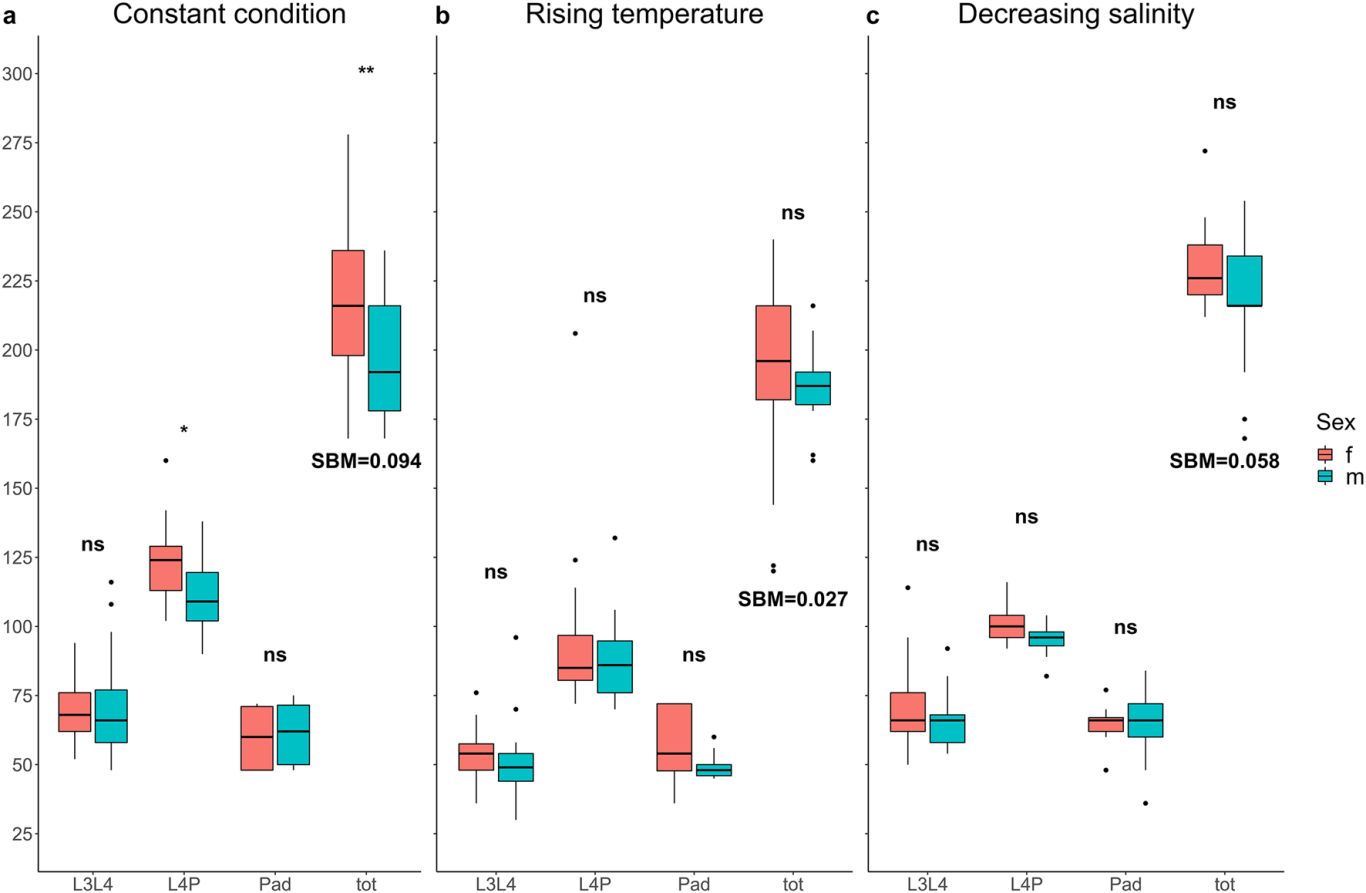
Phenotypic plasticity in development time. Differences in development time between sexes (red = female, blue = male) in (**a**) constant temperature and salinity condition, (**b**) rising temperature condition and (**c**) decreasing salinity condition. Significance levels: *** = *P* < 0.001, ** = *P* < 0.01, * = *P* < 0.05, ns = *P* > 0.05. Boxplots show median values (middle line), interquartile range (box) and the range values including some outliers (dots which extend beyond the min and max of the boxplot). For each treatment, L3-L4 is the development time computed from L3 to L4 larval instars; L4-P is the development time from L4 larval instar to pupal stage; P-Ad is the development time from pupal to adult stages; Total means the development time from L3 larval instar to adult stage.

Multiple comparisons showed that under constant conditions, sexes differed significantly in the development time of fourth instar larvae (t = 3.06, *P* = 0.02). The difference in development time between sexes at this stage led to sexual dimorphism in total development time (L3 – adult emergence interval) (t = 3.56, *P* = 0.004) (Fig. 2a), with males having a more rapid development time than females (i.e. protandry). On the contrary, under temperature and salinity conditions, the difference in development time between females and males was not significant in any immature stages (all *P* > 0.05) (Fig. 2b, c).

By comparing individual development time among treatments, the temperature condition significantly reduced third instar and fourth instar development time in both males and females compared to constant conditions (females: L3-L4, t = 3.98, *P* < 0.001; L4-P, t = 7.46, *P* < 0.001; males: L3-L4, t = 4.57, *P* < 0.001; L4-P, t = 6.05, *P* < 0.001) (Fig. 2). This effect of increasing temperature caused a reduction of total development time in females by 24.73 ± 9.94 h (mean ± SE) corresponding to a reduction of 11.44%, and in males reduced the total development time by 10.85 ± 8.55 h (mean ± SE), corresponding to a reduction of 5.4%. The salinity condition significantly reduced the fourth instar development time of both sexes compared to the constant condition (females: t = 5.64, *P* < 0.001; males: t = 3.86, *P* = 0.001) (Fig. 2), with a reduction in females by 17.64% and in males by 14.1%.

The Sexual Bimaturism Index (SBM index) calculated on total development time showed that the largest difference between sexes was found in mosquitoes reared under constant conditions (constant condition SBM = 0.094; salinity condition SBM = 0.058; temperature condition SMB = 0.025) (Fig. 2).

### Weight

**Figure 3:**
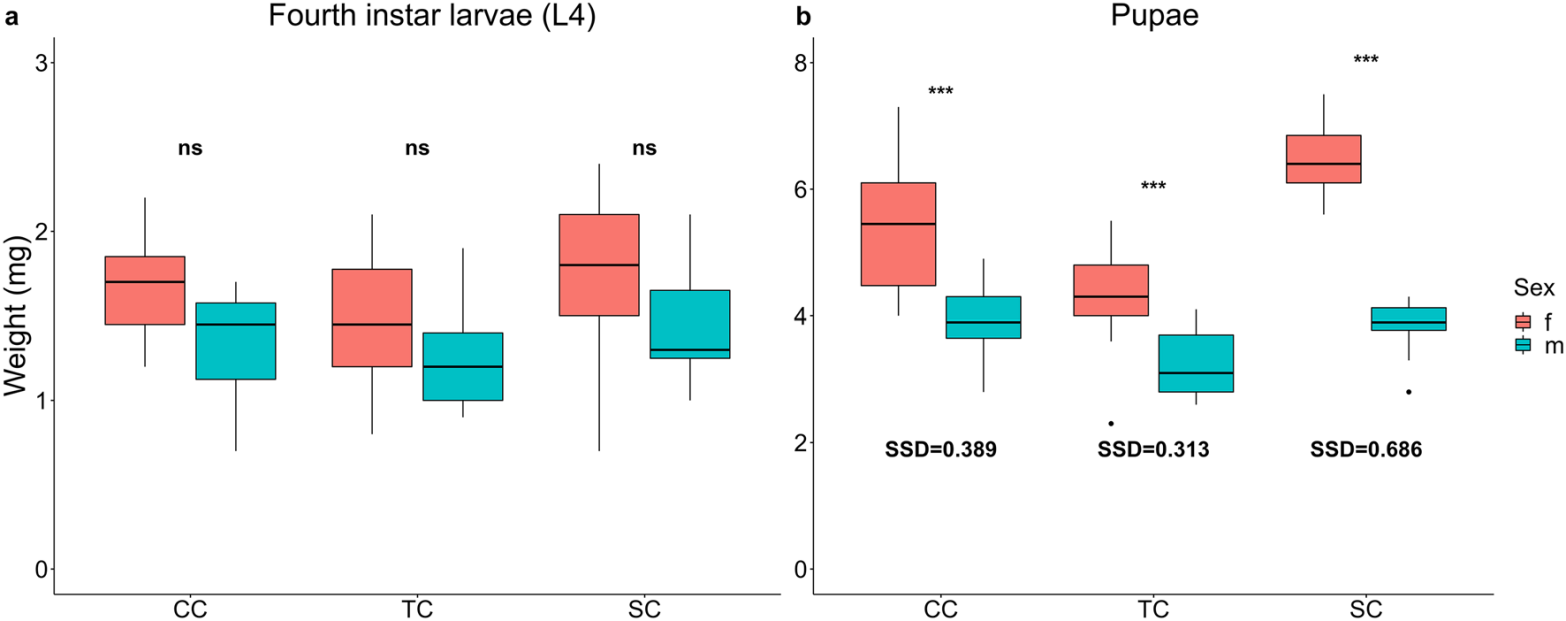
Phenotypic plasticity in larval and pupal weight. Weight differences between sexes (red = female, blue = male) in fourth instar larvae (**a**) and pupae (**b**) in constant temperature and salinity condition (CC), rising temperature condition (TC), and decreasing salinity condition (SC). Significance levels: *** = *P* < 0.001, ** = *P* < 0.01, * = *P* < 0.05, ns = *P* > 0.05. Boxplots show median values (middle line), interquartile range (box) and the range values including some outliers (dots which extend beyond the min and max of the boxplot).

The weight of L4 larval instar and pupae were compared between sexes and treatments using a 3-way ANOVA (Table 1 and Supplementary Table S1). The results showed that the model explains a high percentage of the variance (Adjusted R-squared 0.91). Significant effects of treatment (*F*_*2,172*_ = 47.03, *P* < 0.001), sex (*F*_*1,172*_ = 144.05, *P* < 0.001), stage (*F*_*1,172*_ = 1519.59, *P* < 0.001), and their combinations were observed (Treatment*Sex: *F*_*2,172*_ = 9.44, *P* < 0.001; Treatment*Stage: *F*_*2,172*_ = 25.31, *P* < 0.001; Sex*Stage: *F*_*1,172*_ = 81.54, *P* < 0.001;Treatment*Sex*Stage: *F*_*2,172*_ = 7.17, *P* = 0.001). Multiple comparisons showed that the weight of fourth instar larvae did not differ between sexes under all treatments, while female pupae were significantly heavier than males under each treatment (CC: t = 8.16, *P* < 0.001; TC: t = 6.86, *P* < 0.001; SC: t = 5.93, *P* < 0.001) (Fig. 3a, b).

By comparing individual weights among treatments, rising temperature significantly reduced the pupal weight of females by 1.12 ± 0.46 mg (20.71%) and males by 0.62 ± 0.28 mg (15.94%) compared to constant conditions (females: t = 5.58, *P* < 0.001; males: t = 3.17, *P* = 0.014) (Fig. 3b). On the contrary, decreasing salinity affected only female pupae that were heavier by 1.08 ± 0.37 mg (mean ± SE), corresponding to an increasing of 19.77% compared to pupae developed under constant conditions (females: t = -5.82, *P* < 0.001; males: t = 0.22, *P* > 0.05) (Fig. 3b).

The Sexual Size Dimorphism index (SSD), calculated on pupal weight, in constant conditions was SSD = 0.389; under the temperature condition was SSD = 0.313, while it increased to SSD = 0.686 under the salinity condition (Fig. 3b).

### Morphometry

The morphology of the L4 instar larvae and pupae was analyzed using a principal component analysis (PCA). Then, to assess the effect of sex and treatments, a two-way ANOVA was carried out using the first component of the PCA (Table 1 and Supplementary TableS1).

For the L4 instar larvae, the PCA analysis showed that the first component explains 77.9% of the variance in larval shape (Table 2). The first component had a positive correlation with all the variables and differentiated individuals based on size, with larger values indicating larger larvae. The second component explains an additional 11.6% of the total variance in the data and primarily explains variation in the relative shape of the body. Width measures had a positive correlation with the second component, and length measures had a negative correlation, discriminating individuals based on their stumpiness (Table 2).

**Table 2:** PCA analysis. Results of the principal component analysis on morphometric measures of fourth instar larvae and pupae.

	Eigenvalues	PC1	PC2	PC3	PC4	PC5	PC6
4th instar larvae							
Proportion of total variance explained		0.779	0.116	0.039	0.036	0.014	0.012
	Head width	0.381	0.551	0.497	0.420	0.235	0.269
	Thorax width	0.437	0.227	–	− 0.297	0.191	− 0.795
	Abdomen width	0.410	0.389	− 0.392	− 0.439	− 0.419	0.398
	Thorax length	0.384	− 0.541	0.425	− 0.475	0.255	0.297
	Abdomen length	0.415	− 0.264	− 0.627	0.387	0.450	0.114
	Total length	0.419	− 0.362	0.159	0.409	− 0.682	− 0.189
Pupae							
Proportion of total variance explained		0.970	0.029	–	–	–	–
	Cephalo-thorax width	0.707	0.707	–	–	–	–
	Cephalo-thorax length	0.707	**-**0.707	–	–	–	–

**Figure 4:**
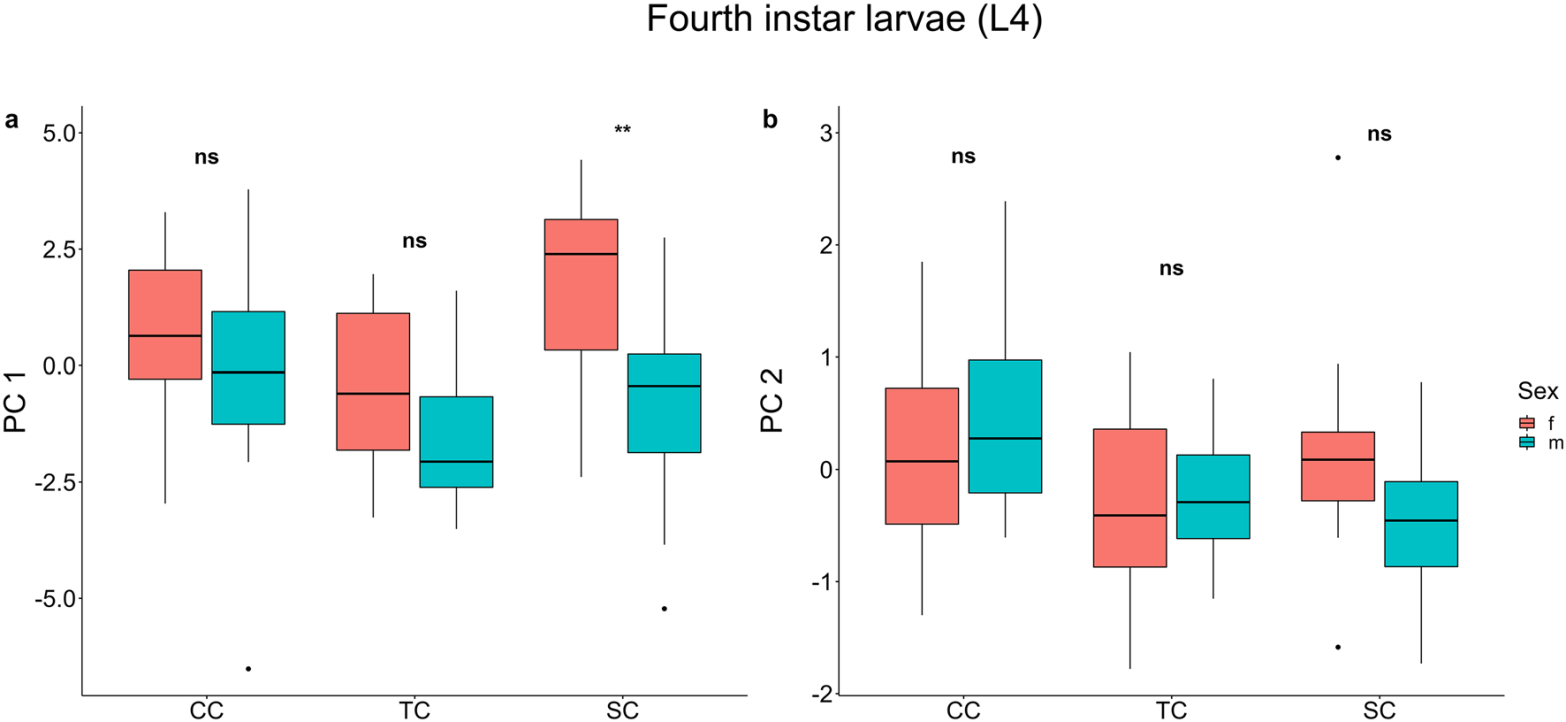
Phenotypic plasticity in larval morphological traits. First and second components of PCA analysis on morphometric measures of fourth instar larvae (**a**, **b**). Differences between sexes (red = female, blue = male) in constant temperature and salinity condition (CC), rising temperature condition (TC), and decreasing salinity condition (SC). Significance levels: *** = *P* < 0.001, ** = *P* < 0.01, * = *P* < 0.05, ns = *P* > 0.05. Boxplots show median values (middle line), interquartile range (box) and the range values including some outliers (dots which extend beyond the min and max of the boxplot).

The two-way ANOVA carried out using the first component of the PCA showed a significant effect of treatment (*F*_*2,85*_ = 6.20, *P* = 0.003) and sex (*F*_*1,85*_ = 14.17, *P* < 0.001) on the larval morphology (Table 1 and Supplementary Table S1). (Fig. 4a). Multiple comparisons showed non-significant differences between treatments and between sexes except for the salinity condition where females were overall bigger than males (t = 3.56, *P* = 0.004). The two-way ANOVA carried out using the second component of the PCA showed a significant effect of treatments (*F*_*2,85*_ = 3.97, *P* = 0.002) on fourth instar larvae morphology. Multiple comparisons showed non-significant differences between sex and between treatments, except for the salinity condition that significantly affected larval males, making them more stumpy related to males developed under constant conditions (t = 2.97, *P* = 0.024) (Fig. 4).

**Figure 5:**
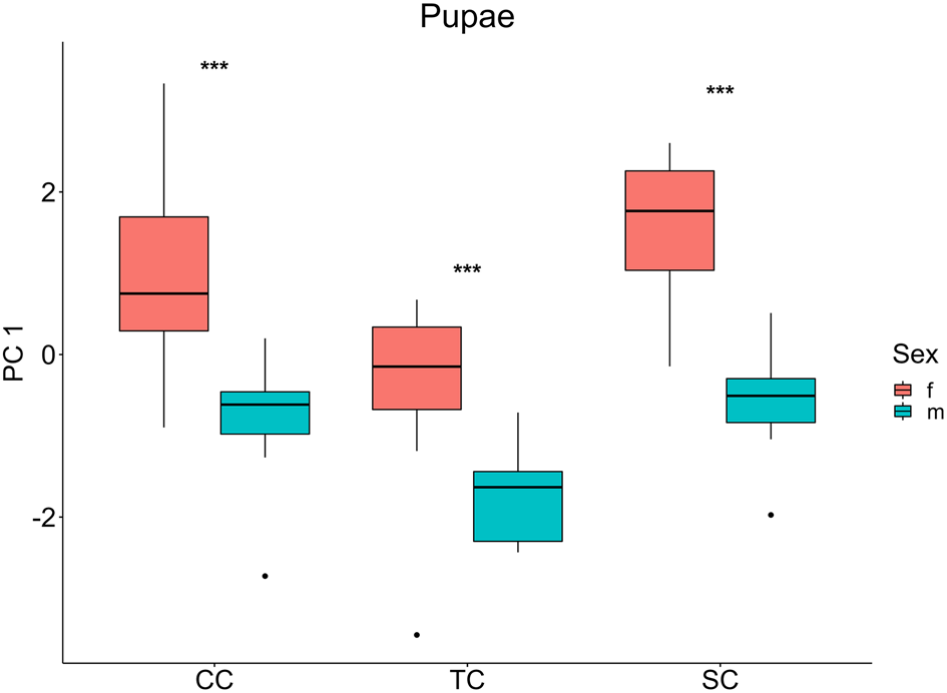
Phenotypic plasticity in pupal morphological traits. First component of PCA analysis on morphometric measures of pupae in constant temperature and salinity condition (CC), rising temperature condition (TC), and decreasing salinity condition (SC). Significance levels: *** = *P* < 0.001, ** = *P* < 0.01, * = *P* < 0.05, ns = *P* > 0.05. Boxplots show median values (middle line), interquartile range (box) and the range values including some outliers (dots which extend beyond the min and max of the boxplot).

The first component of the PCA analysis on pupal morphology explained 97.08% of the total variance. Length and width were positively correlated to the first component, discriminating the individuals on their overall dimension. No differences in shape were found in pupae (Table 2). The two-way ANOVA carried out using the first component of the PCA showed a significant effect of treatments (*F*_*2,86*_ = 36.49, *P* < 0.001) and sex (*F*_*1,86*_ = 108.28, *P* < 0.001) on pupal morphology (Fig. 5). Multiple comparisons revealed significant differences between sexes under each developmental condition, with males smaller than females (CC: t = 6.25, *P* < 0.00; TC: t = 4.09, *P* < 0.001; SC: t = 7.53, *P* < 0.001). Furthermore, rising temperature during development led to reduced pupal size in both sexes (females: t = 4.54, *P* < 0.00; males: t = 3.29, *P* = 0.009) (Fig. 5). SSD index calculated on cephalo-thorax length was 0.124, 0.092 and 0.142 under constant, temperature and salinity conditions, respectively.

## Discussion

In insects, sexes frequently differ in plastic response to environmental variations such as food limitation, food quality, and larval density, with the larger sex—typically females—displaying a stronger response than the smaller sex^9,19,22^. Although temperature-induced plastic responses have received much attention, differences in thermal plasticity between sexes are still largely debated^20,35–37^. Teder et al. (2022), through a meta-analysis conducted on several insect species, found that sexes differ significantly in their plastic response to diet while temperature-induced plastic responses were systematically less pronounced. They pointed out the existence of substantial physiological constraints on the evolvability of thermal reaction norms in insects. At the same time, there are evidence of sexual differences in plastic response to temperature^21,26,38^. For example, in a population of the butterfly *Pararge aegeria* was observed that sexual differences varied in both direction and strength over a range of increasing temperatures^8^. Here, we found that increasing temperature caused a reduction in development time in both males and females, but the females' reduction was more pronounced, exhibiting greater phenotypic plasticity. A similar pattern of increased thermal responsiveness in females was observed for body mass and size, leading to a greater reduction in females than in males.

Contrary to temperature, the effect of salinity on mosquitoes' development has been scarcely investigated. Some previous studies found that salinity caused a reduction or a prolonged larval development, depending on the species, and also for the same species, results obtained are in contrast^39–41^. We observed that decreasing salinity didn't significantly affect development time, but it triggered a plastic response only in females' body size, causing an increase in body mass.

The association between development time and size is a typical component of life-history models (Roff 1992, Stearns 1992) and the association of dimorphism in these traits is a common pattern across species, especially in insects. For example, Teder et al. (2021) conducting a meta-analysis on 192 insect species found a strong association between direction and degree of sexual bimaturism and SSD with larger sex having longer development time. Our results are consistent with these observations showing that females are bigger and have a longer development time than males in control treatment, confirming the association between female biased SSD and protandry. However, we found that this association is condition dependent. In fact, due to the differential expression of plasticity between sexes, mosquito males and females emerge simultaneously under variable temperature and salinity conditions, but females reach a larger size and mass anyway, implying that differences in plastic response between sexes and between traits break up the link between sexual bimaturism and sexual size dimorphism.

Consequently, the hypotheses proposed to date for the association between sexual bimaturism and sexual size dimorphism do not help explain patterns that emerged from our data. Indeed, according to the mating opportunity hypothesis, the advantage conferred to males in terms of mating probability by the faster development should keep constant the absolute difference in development time between males and females—being selected per se—and thus should be insensitive to environmental conditions^8^. Our results show that the degree of sexual dimorphism in development time could vary under variable temperature and salinity conditions due to differential plasticity between the sexes, providing no support for this first hypothesis. Moreover, since the degree of protandry and sexual size dimorphism vary independently between environments, it seems unlikely that the difference in development time could be a simple by-product of selection for large female size, as predicted by the constraint hypothesis.

In recent years, compelling empirical evidence showed a positive correlation between sex-specific plasticity of a trait and sexual dimorphism in the same trait, supporting the idea that condition dependence plays a pivotal role in the evolution of sexual size dimorphis^19,21–23^. Our results extend the importance of environmental conditions to the evolution of association and covariation of multiple traits. Taking into account differential plasticity between sexes, protandry or its absence might co-occur within insect populations independently with any type of sexual size dimorphism^13^. Especially in insects, where phenotypic plasticity in traits such as development time and body size is largely diffused, studying the relation between sexual bimaturism and sexual size dimorphism without considering environmental effects might have led to a limited view of an extremely complex scenario.

## Methods

### Sampling and experimental conditions

Experiments were carried out on individuals of *Ae. mariae* obtained from eggs collected in July 2021 from multiple supralittoral rock pools of San Felice Circeo, Italy (41°13′18.77″ N, 13° 4′5.51″ E). We designed three different experimental treatments by changing temperature and salinity conditions and measured development time and body size during larval and pupal development (Fig. 1). In the first treatment, individuals were maintained at constant conditions of temperature (26 ±  °C) and salinity (50‰, 50 g/l) throughout the experiment (hereafter, constant condition, CC). In the second treatment, individuals were exposed to rising temperature (hereafter temperature condition, TC) as follows: third instar larvae were maintained at 29 °C; fourth instar larvae at 31 °C, and pupae at 34 °C. In the third treatment, individuals were exposed to decreasing salinity (hereafter salinity condition, SC) from 50‰ to 0‰. Twenty-four hours after reaching the third instar, saline water was replaced in each cup with fresh water at intervals of two hours until the salinity reached 0‰.

To achieve controlled and uniform temperature variations, the experimental cups were partially submerged in tanks containing 75 l of water, in which two thermostats were used for temperature manipulation.

### Phenotypic traits measurements

Development time, weight, and morphological traits were measured for larval and pupal stages. To accurately measure these traits, all individuals had to be of the same age at the start of the experiment. At this aim, we adopted the following strategy: L1 instar (< 24 h) were selected and placed in plastic trays (30 × 30 × 15 cm) filled with water from the sampling rock pools. When the L1 larvae reached the second instar, they were individually (N = 210) placed into individual 300 ml plastic cups filled with 100 ml of tap water, previously salted with aquarium salt (Tetra Marine Seasalt). Then, we checked every two hours along the 24 h to find the L2 exuvia and started the experiment once the larvae reached the third instar. In this way, we synchronized the beginning of the experiments for each larva with a maximum error of two hours. Once the larvae reached the third instar they were randomly placed into developmental treatments (N = 70 × treatment), and trait measures started (Fig. 1).

To gain reliable estimates of development time, each plastic cup was monitored every 2 h (day and night) until the emergence of adults. We recorded the date and time of the larval exuvia spotting, larval-pupal ecdysis, and adult emergence. Likewise, to gain reliable estimates of body weight, the weights of fourth instars and pupae were recorded to the nearest 0.1 mg within two hours from the ecdysis using a precision balance (Ohaus PRseries PR224). Similarly, morphometric measures were obtained by taking digital pictures using stereomicroscope Leica EZ4W at magnification 1 × of all individuals within two hours from the ecdysis and taking measurements with the open-access software IMAGEJ. The length and width of the thorax and abdomen and the total body length were measured for larvae. Cephalo-thorax length and width were measured for pupae (Supplementary Fig. S1)^42,43^. The sex of individuals was determined at the pupal stage by checking the last cephalo-thorax segment (Supplementary Fig. S1). After the record of weight and morphometric traits, a random sample of L4 and pupae (10–12 individuals for each stage) were removed and placed in RNAlater for future molecular analyses.

The experiments were run in a climate chamber set to 26 ± 1 °C, 14 h light and 10 h dark regime. Experimental larvae were fed daily with 1 mg of cat food. Because of water evaporation, the volume of each cup was checked daily, and prewarmed water was added as needed to maintain a total volume of 100 ml.

### Statistical analysis

Development time and weight data were analyzed using a three-way ANOVA. Sex, treatment, developmental stage, and their combined effects were used as independent variables, and multiple comparisons were carried out using *glht* function implemented in *multcomp* R-package^44^*.* Morphometric data of larvae and pupae were analyzed separately by principal component analysis (PCA). Subsequently, the effect of sex and treatment and their combined effect was tested by performing a two-way ANOVA on the first PCA components. Multiple comparisons were carried out using *glht* function implemented in *multcomp* R-package^44^*.* The degree of sexual dimorphism in development time was calculated with the Sexual Bimaturism index as SBM = (development time of the sex with longer development time/ development time of the sex with shorter development time)−1 ^9^. The analogous Sexual Size Dimorphism index as SSD = (larger sex/smaller sex)-1 was calculated to estimate sexual size dimorphism^45^. Since the PCA analysis did not highlight shape differences between pupae (see Results), for morphometric measures we calculated SSD index using the cephalo-thorax length. All statistical analyses were performed in R vers.4.1.2 (http://www.Rproject.org/).

### Supplementary Information

Supplementary Information.

## Data Availability

The datasets generated and analysed during the current study are available from the corresponding author on reasonable request.
