# Supplementary material for: Sex-biased phenotypic plasticity affects sexual dimorphism patterns under changing environmental conditions
Source: Sci Rep. 2024 Jan 9;14:892. doi: 10.1038/s41598-024-51204-6 (PMC10776787; doi:10.1038/s41598-024-51204-6)
Supplement: Supplementary file 1 — Supplementary Information. [file 41598_2024_51204_MOESM1_ESM.pdf]

Sex-biased phenotypic plasticity affects sexual dimorphism patterns under changing environmental conditions

Author names: Giulia Cordeschi[1], Daniele Canestrelli[2], Daniele Porretta[1]

Affiliations:

[1] Department of Environmental Biology, Sapienza University of Rome, Via dei Sardi 70, Rome, Italy

[2] Department of Biology and Ecology, Tuscia University, Largo dell'Università s.n.c., Viterbo, Italy

Corresponding author email address: daniele.porretta@uniroma1.it

Supporting information

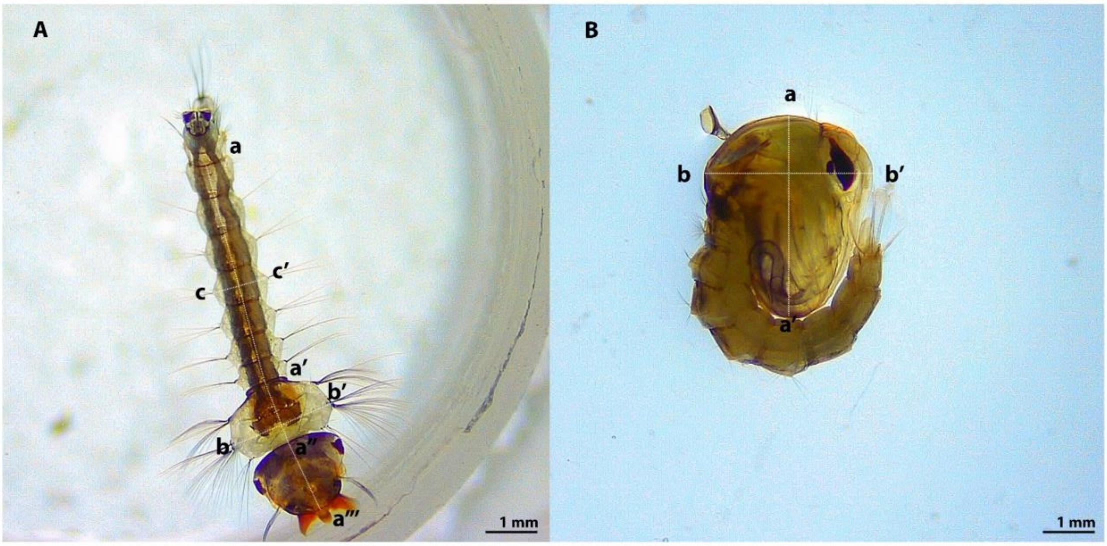

Supplementary Figure S1. Marks used in morphometric analyses for larval (L4) (A) and pupal (B) stages of *Aedes mariae*. A: Aa’: Abdomen length; a’a”: Thorax length; aa”’: Total length; bb’: Thorax width; cc’: Abdomen width. **B**: aa’: Cephalo-thorax length; bb’: Cephalo-thorax width. (Photo Giulia Cordeschi)

Supplementary Table 1: Results of ANOVA. Effects of treatment, sex and developmental stage on development time, weight, and first PCA components of morphometric measures of larvae and pupae.

		**df**	**SS**	***F***	**Pr > *F***
**Development time**	Treatment	2,599	**31656**	**64.24**	**< 0.001**
	Sex	1,599	**4741**	**19.24**	**< 0.001**
	Stage	3,599	**1838362**	**2487.22**	**< 0.001**
	Treatment * Sex	2,599	147	0.2987	0.741
	Treatment * Stage	6,599	**20492**	**13.86**	**< 0.001**
	Sex * Stage	3,599	**2369**	**3.20**	**0.022**
	Treatment * Sex * Stage	6,599	1321	0.89	0.499
**Weight**	Treatment	2,172	**28.2**	**47.03**	**< 0.001**
	Sex	1,172	**43.2**	**144.05**	**< 0.001**
	Stage	1,172	**455.6**	**1519.59**	**< 0.001**
	Treatment * Sex	2,172	**5.7**	**9.44**	**< 0.001**
	Treatment * Stage	2,172	**15.2**	**25.31**	**< 0.001**
	Sex * Stage	1,172	**24.4**	**81.54**	**< 0.001**
	Treatment * Sex * Stage	2,172	**4.3**	**7.17**	**0.001**
**Morphometry larvae – 1 PCA component**	Treatment	2,85	**45.43**	**6.20**	**0.003**
	Sex	1,85	**51.86**	**14.17**	**0.0003**
	Treatment * Sex	2,85	11.60	1.58	0.21
**Morphometry larvae – 2 PCA component**	Treatment	2,85	**5.05**	**3.97**	**0.02**
	Sex	1,85	0.00	0.01	0.93
	Treatment * Sex	2,85	3.69	2.90	0.06
**Morphometry pupae – 1 PCA component**	Treatment	2,86	**47.11**	**36.49**	**< 0.001**
	Sex	1,86	**69.89**	**108.28**	**< 0.001**
	Treatment * Sex	2,86	2.89	2.24	0.11
